# Supplementary material for: Externalized Reusable Permanent Pacemaker for Prolonged Temporary Cardiac Pacing in Critical Cardiac Care Units: An Observational Monocentric Retrospective Study
Source: J Clin Med. 2022 Dec 4;11(23):7206. doi: 10.3390/jcm11237206 (PMC9736961; doi:10.3390/jcm11237206)
Supplement: Supplementary file 1 [file jcm-11-07206-s001.zip › jcm-2072402-supplementary.pdf]

Supplementary Appendix

Externalized reusable permanent pacemaker for prolonged temporary pacing in critical cardiac care units: an observational monocentric retrospective study

Authors: Maxime Beneyto (1); Matthieu Seguret (2); Marine Taranzano (2); Pierre Mondoly (1); Caroline Biendel (2); Anne Rollin (1); Fanny Bounes (3); Michel Galinier (2); Meyer Elbaz (2); Philippe Maury (1); and Clément Delmas (2)

Affiliations:

(1) Electrophysiology and pacing department, Rangueil University Hospital, Toulouse, France

(2) Intensive cardiac care unit, Rangueil University Hospital, Toulouse, France

(3) Anaesthesiology and Critical Care Unit, Toulouse University Hospital, 31400 Toulouse, France; INSERM U1297, Paul Sabatier University, Toulouse, France

Table of Contents

Supplementary Data 2

Supplementary Table S1. Population characteristics according to PTCP indication 4

Supplementary Table S2. Outcomes according to temporary cardiac pacing indication........ 7

Supplementary Table S3. Outcomes according to the occurrence of complications. 8

Supplementary Data

Collected variables

Demographic and clinical data: age, sex, unit of admission, invasive mechanical ventilation at implantation, renal replacement therapy at implantation, SAPS II at implantation, PTCP indication, PTCP implantation method, PTCP device type, PTCP vacular access, delay from admission to PTCP implantation, and PTCP duration.

Medications at PTCP implantation: vitamin K antagonists, non-vitamin K antagonist oral anticoagulation, aspirin, P2Y12 receptor inhibitor, unfractionated heparin, low molecular weight heparin, betablocker, non-dihydropyridine calcium channel blocker, amiodarone, other anti-arrhythmic drugs, catecholamines, and antibiotics

Cardiovascular risk factors: obesity, diabetes mellitus, arterial hypertension, tobacco, dyslipidemia

Medical history: peripheral artery disease, immunosuppression, respiratory insufficiency, chronic kidney disease, cancer, prior heart disease (ischemic heart disease, dilated cardiomyopathy, rhythm disorder, valvular heart disease, or other)

Arrhythmias

The 27th patient developed an atrial flutter during PTCP. He had septic arthritis associated with diabetic ketoacidosis and 2:1 atrioventricular block.

The 44th patient developed atrial fibrillation during PTCP. She had a CIED infection associated with endocarditis and spondylodiscitis.

The 18th patient exhibited ventricular tachycardia during PTCP. He presented with ST-segment elevation myocardial infarction 10 hours after the onset of chest pain. The infarction-related artery thrombus could not be crossed, impeding revascularization. A week later, he developed an electrical storm, requiring ablation; the latter led to complete atrioventricular block, which motivated the use of PTCP. Episodes of sustained slow ventricular tachycardia reoccurred afterward.

The 30th patient exhibited ventricular fibrillation during PTCP. He presented with aborted cardiac arrest caused by an ST-segment elevation myocardial infarction which had been lasting for 48 hours. He developed numerous runs of non-sustained ventricular tachycardia early on. He experienced repeated nursing-associated vagal bradycardia inducing hemodynamic instability,

which motivated the use of PTCP. A month and a half after admission, the patient exhibited several episodes of ventricular fibrillation initiated by short-coupled premature ventricular complexes.

The 36th patient exhibited ventricular tachycardia during PTCP. He presented with aborted cardiac arrest caused by an ST-segment elevation myocardial infarction. He experienced repeated nursing-associated vagal bradycardia inducing hemodynamic instability, which motivated the use of PTCP. New episodes of ventricular tachycardia occurred twelve days after admission.

The 43rd patient exhibited ventricular tachycardia during PTCP. She presented with ST-segment elevation myocardial infarction 10 hours after chest pain onset, complicated with cardiogenic shock and ventricular tachycardia. She rapidly developed complete atrioventricular block, which motivated the use of PTCP. Her hemodynamic status deteriorated again afterward with another cardiogenic shock and new episodes of sustained ventricular tachycardia, eventually leading to treatment withdrawal and death.

Causes of death after hospital discharge

The 34th patient was admitted for intracranial hemorrhage; his hospitalization was complicated by pulmonary sepsis and complete atrioventricular block, indicating the use of PTCP. Several episodes of ventilation-acquired pneumonia had occurred, the last identified bacteria being *Acinetobacter baumanii* and *Enterobacter cloacae*. During the six following months, several new infectious episodes occurred, among which *Enterobacter cloacae* pneumonia and methicillin-resistant *Staphylococcus epidermidis* bacteremia. These repeated infections eventually lead to multiorgan failure and death.

The 42nd patient was admitted for *Candida albicans* pneumonia associated with complete atrioventricular block, without evidence of endocarditis. His past medical history comprised hemodialysis and cirrhosis. He underwent PTCP then leadless pacemaker implantation two weeks later. During the following month, he developed an intracranial abscess complicated with hepatic encephalopathy and seizure. He died after treatment withholdal.

Supplementary Table S1. Population characteristics according to PTCP indication

Characteristic	Potentially transient high-grade conduction disorder (N = 17)	Pacemaker indication but ongoing infection (N = 14)	Pacing-dependent patient with CIED infection (N = 7)	Nursing-associated vagal bradycardia (N = 7)	Recurrent sustained VT (N = 1)	p[a]
Age (years)	71 (62 – 76)	73 (71 – 80)	84 (77 – 88)	59 (47 – 67)	55	0.39
Female sex	7 (41.2)	4 (28.6)	4 (57.1)	1 (14.3)	1 (100)	0.52
Unit of admission						0.30
Critical care	17 (100)	14 (100)	3 (43)	7 (100)	1 (100)	
Cardiology ward	0 (0)	0 (0)	4 (57)	0 (0)	0 (0)	
Number of cardiovascular risk factors	1 (1 – 2)	2 (1 – 3)	2 (2 – 3)	2 (1 – 2)	1	0.24
Obesity	5 (29.4)	6 (42.9)	2 (28.6)	1 (14.3)	0 (0)	0.30
Diabetes mellitus	4 (23.5)	5 (35.7)	2 (28.6)	1 (14.3)	0 (0)	0.47
Arterial hypertension	8 (47.1)	6 (42.9)	4 (57.1)	2 (28.6)	1 (100)	>0.99
Tobacco	4 (23.5)	6 (42.9)	3 (42.9)	5 (71.4)	0 (0)	0.75

Dyslipidemia	3 (17.6)	7 (50)	3 (42.9)	2 (28.6)	0 (0)	0.17
Peripheral artery disease	1 (5.9)	2 (14.3)	2 (28.6)	1 (14.3)	0 (0)	>0.99
Respiratory insufficiency	1 (5.9)	2 (14.3)	2 (28.6)	1 (14.3)	0 (0)	>0.99
Chronic kidney disease	1 (5.9)	4 (28.6)	2 (28.6)	2 (28.6)	0 (0)	0.42
Prior heart disease	9 (52.9)	12 (85.7)	7 (100)	0 (0)	1 (100)	<0.05
Ischemic heart disease	6 (66.7)	4 (33.3)	4 (57.1)	—	0 (0)	0.26
Dilated cardiomyopathy	0 (0)	0 (0)	0 (0)	—	1 (100)	>0.99
Rhythm disorder	3 (33.3)	8 (66.7)	5 (71.4)	—	1 (100)	0.70
Valvular heart disease	1 (11.1)	10 (83.3)	2 (28.6)	—	0 (0)	<0.001
Other	2 (22.2)	0 (0)	0 (0)	—	0 (0)	0.50
Medications at implantation						
Vitamin K antagonists	3 (17.6)	2 (14.3)	0 (0)	0 (0)	0 (0)	0.63
Non-vitamin K antagonist oral anticoagulation	0 (0)	2 (14.3)	1 (14.3)	0 (0)	0 (0)	0.22
Aspirin	13 (76.5)	6 (42.9)	4 (57.1)	4 (57.1)	0 (0)	0.20
$P2Y_{12}$ receptor inhibitor	8 (47.1)	1 (7.1)	1 (14.3)	3 (42.9)	0 (0)	0.07
Catecholamines	4 (23.5)	7 (50)	1 (14.3)	6 (85.7)	1 (100)	0.52

Antibiotics	5 (29.4)	14 (100)	6 (85.7)	6 (85.7)	1 (100)	<0.01
Invasive mechanical ventilation at implantation	2 (11.8)	4 (28.6)	0 (0)	4 (57.1)	1 (100)	0.71
Renal replacement therapy at implantation	3 (17.6)	3 (21.4)	2 (28.6)	1 (14.3)	0 (0)	>0.99
SAPS II at implantation	33 (27 – 39)	43 (30 – 56)	30 (25 – 41)	51 (40 – 77)	54	0.46

[a] *p-value for the comparison of patients with pacemaker indication but ongoing infection vs all other patients.*

CIED, cardiac implantable electronic device; PTCP, prolonged temporary cardiac pacing; SAPS II, Simplified Acute Physiology Score II; VT, ventricular tachycardia.

Supplementary Table S2. Outcomes according to temporary cardiac pacing indication.

Indication	In-hospital outcomes						Six-month outcomes				
	N	Complication	Removal	Definite device implantation	Death	Missing	N	Device dys-function	Device infection	Death	Missing
Potentially transient high-grade conduction disorder	17	4 (24)	10 (59)	5 (29)	2 (12)	1 (6)	15	0 (0)	0 (0)	0 (0)	1 (7)
Pacemaker indication but ongoing infection	14	3 (21)	0 (0)	13 (93)	1 (7)	0 (0)	13	0 (0)	2 (15)	1 (8)	1 (8)
Pacing-dependent patient with CIED infection	7	1 (14)	0 (0)	7 (100)	0 (0)	0 (0)	7	0 (0)	0 (0)	0 (0)	1 (14)
Nursing-associated vagal bradycardia	7	4 (57)	4 (57)	1 (14)	2 (29)	0 (0)	5	0 (0)	0 (0)	0 (0)	0 (0)
Recurrent sustained ven-tricular tachycardia	1	0 (0)	1 (100)	0 (0)	0 (0)	0 (0)	1	—	—	0 (0)	0 (0)

CIED, cardiac implantable electronic device

Supplementary Table S3. Outcomes according to the occurrence of complications.

	With PTCP-associated complication	Without PTCP-associated complication
**In-hospital outcomes**	**N = 12**	**N = 34**
Removal	4 (33)	11 (32)
Definite device implantation	5 (42)	21 (62)
Death	3 (25)	2 (6)
**Six-month outcomes**	**N = 9**	**N = 32**
Device dysfunction	0 (0)	0 (0)
Device infection	0 (0)	2 (6)
Death	0 (0)	1 (3)
Missing	1 (11)	2 (6)

PTCP, prolonged temporary cardiac pacing
